# Dynamic Expression Patterns of Differential Proteins during Early Invasion of Hepatocellular Carcinoma

**DOI:** 10.1371/journal.pone.0088543

**Published:** 2014-03-10

**Authors:** Rong-Xin Chen, Hai-Yan Song, Yin-Ying Dong, Chao Hu, Qiong-Dan Zheng, Tong-Chun Xue, Xiao-Hui Liu, Yang Zhang, Jie Chen, Zheng-Gang Ren, Yin-Kun Liu, Jie-Feng Cui

**Affiliations:** 1 Liver Cancer Institute, Zhongshan Hospital, Fudan University & Key Laboratory of Carcinogenesis and Cancer Invasion, Ministry of Education, Shanghai, PR China; 2 Institute of Digestive Disease, Longhua Hospital, Shanghai University of Traditional Chinese Medicine, Shanghai, China; 3 Renji Hospital, School of Medicine, Shanghai Jiao Tong University, Shanghai, PR China; 4 Institute of Biomedical Science, Fudan University, Shanghai, PR China; University of Navarra School of Medicine and Center for Applied Medical Research (CIMA), Spain

## Abstract

**Background:**

Tumor cell invasion into the surrounding matrix has been well documented as an early event of metastasis occurrence. However, the dynamic expression patterns of proteins during early invasion of hepatocellular carcinoma (HCC) are largely unknown. Using a three-dimensional HCC invasion culture model established previously, we investigated the dynamic expression patterns of identified proteins during early invasion of HCC.

**Materials and Methods:**

Highly metastatic MHCC97H cells and a liver tissue fragment were long-term co-cultured in a rotating wall vessel (RWV) bioreactor to simulate different pathological states of HCC invasion. The established spherical co-cultures were collected on days 0, 5, 10, and 15 for dynamic expression pattern analysis. Significantly different proteins among spheroids at different time points were screened and identified using quantitative proteomics of iTRAQ labeling coupled with LC–MS/MS. Dynamic expression patterns of differential proteins were further categorized by K-means clustering. The expression modes of several differentially expressed proteins were confirmed by Western blot and qRT–PCR.

**Results:**

Time course analysis of invasion/metastasis gene expressions (MMP2, MMP7, MMP9, CD44, SPP1, CXCR4, CXCL12, and CDH1) showed remarkable, dynamic alterations during the invasion process of HCC. A total of 1,028 proteins were identified in spherical co-cultures collected at different time points by quantitative proteomics. Among these proteins, 529 common differential proteins related to HCC invasion were clustered into 25 types of expression patterns. Some proteins displayed significant dynamic alterations during the early invasion process of HCC, such as upregulation at the early invasion stage and downregulation at the late invasion stage (e.g., MAPRE1, PHB2, cathepsin D, etc.) or continuous upregulation during the entire invasion process (e.g., vitronectin, Met, clusterin, ICAM1, GSN, etc.).

**Conclusions:**

Dynamic expression patterns of candidate proteins during the early invasion process of HCC facilitate the discovery of new molecular targets for early intervention to prevent HCC invasion and metastasis.

## Introduction

Metastasis and recurrence are major obstacles towards to a significant improvement in HCC treatment or HCC prognosis after surgical resection [1]. In recent years, increasing evidence on metastasis has suggested that multiple factors, such as malignant phenotypes of cancer cells, extracellular matrix, immunity, angiogenesis, or target organs, are all involved in this complicated pathological process [2], thereby rendering great difficulties in the design of *in vitro* simulation experiments for HCC metastasis explaining. The development of experimental models has considerably contributed to our understanding of the pathogenesis of HCC metastasis [3]. However, no available *in vitro* experimental model can yet mirror the exact pathological progression of HCC metastasis. As well, single experimental models are known to mimic only a subclass of cancer or one of its pathological phases [4].

Exclusive reliance on data from traditional stepwise metastatic HCC cells, HCC animal models, and clinical cancer tissue samples results in difficulties in understanding the diverse pathological changes that occur during HCC metastasis. In addition, most of the known metastasis-associated proteins/genes are identified by comparative proteomics/genomics between primary tumor tissues and metastases, primary tumors with and without metastasis, cancer tissues and paracancerous tissues, as well as HCC cells with different metastasis potentials [5]–[8]. Samples obtained “after metastasis” have been used to demonstrate that key events and molecules altered at early invasion stage of metastasis may easily be missed. Expression patterns of molecules responsible for early invasion may also differ from those of known and identified molecules associated with metastasis. Thus, establishing a novel model clearly mimicking HCC invasion processes can help discover “root” molecules in the early invasion of HCC and ultimately find potential therapeutic targets for early intervention of cancer invasion and metastasis.

A three-dimensional (3D) culture model reflects the clinical pathological characteristics of solid tumors more accurately than two-dimensional (2D) culture models [9], [10]. Tumor spheroids exhibit many distinct advantages in resembling the 3D cytoarchitecture and pathophysiological micromilieu of tumors [11]. The established 3D hepatocyte cultures in some studies show better cell structure and liver-specific functions [12]. HCC spheroids also exhibit the most malignant properties of HCC tumors [13]. However, little is known about the pathological changes of early invasion of HCC cells in the 3D state. Using a 3D HCC invasion culture model established previously, we identified several candidate proteins involved in early invasion of HCC using quantitative proteomics by isobaric tags for relative and absolute quantitation (iTRAQ) labeling coupled with liquid chromatography-tandem mass spectrometry (LC–MS/MS) and explored their dynamic expression patterns.

## Materials and Methods

### Cell culture and preparation of liver tissue fragments

Highly metastatic MHCC97H cells, established at the Liver Cancer Institute of Fudan University [14], [15], were cultured in standard DMEM/F12 medium (GIBCO, USA) supplemented with 10% fetal bovine serum (Biowest, South America Origin) and 1% penicillin–streptomycin (100 unit/mL each; GIBCO). The culture medium was changed twice weekly. When cells had grown to 90% confluence, they were harvested for 3D co-culture. Male athymic BALB/C–nu/nu nude mice (4 weeks old) were obtained from Shanghai SLAC Laboratory Animal Co. Ltd. Animal care and study protocols were in accordance with guidelines established by the Shanghai Medical Experimental Animal Care Committee. The study protocol was approved by the Committee on the Ethics of Animal Experiments of Zhongshan Hospital, Fudan University. Nude mice were executed by dislocation, and fresh livers were carefully excised by operation. Blood in the liver tissue was removed by repeated washing with 0.9% normal saline. Liver tissue fragments (2 mm×2 mm×2 mm) were prepared and pre-incubated with DMEM/F12 culture medium until use.

### 3D co-culture of HCC cells and a liver tissue fragment in RWV bioreactor

A 3D co-culture in a rotating wall vessel (RWV) bioreactor was performed as described in our previous study [13], [16]. Briefly, approximately 1×10^7^ MHCC97H cells suspended in 10 mL of DMEM/F12 medium and a liver tissue fragment were transferred into a RWV bioreactor (Synthecon, Houston, TX, USA) for long-term rotating co-culture. After initial culture conditions of low-speed rotation for 24 h, a co-culture spheroid was formed. The spheroid was then maintained in a freely suspended state within the vessel by modulation of its speed. The medium was replaced after 36 h. Spheroids were collected at different time points (0, 5, 10, and 15 d after co-culture).

### Quantitative reverse transcription polymerase chain reaction (qRT–PCR)

Total RNA of the collected spheroids were extracted using TRIZOL (Invitrogen, USA) according to the manufacturer's protocol and 2 µg of it was reverse-transcribed into cDNA with the primer oligo(dT)_18_ using a RevertAid first-strand cDNA synthesis kit (Fermentas). cDNA was then used as template for PCR amplification of specific genes (SYBR Green PCR Master Mix Kit, Invitrogen). Relative expressions of genes were normalized to GAPDH and reported as 2^−Δ*CT*^, and Δ*CT* = *Ct*(target gene)−*Ct*(GAPDH). The primer sequences were as follows: CXCL12 sense, 5′-GTT CAA AGC CAG CGT C-3′; CXCL12 antisense, 5′-TAG TTC ACC CCA AAG GA-3′; MMP2 sense, 5′-GTT CAT TTG GCG GAC TGT-3′; MMP2 antisense, 5′-AGG GTG CTG GCT GAG TAG-3′; MMP9 sense, 5′-CTT TGG ACA CGC ACG AC-3′; MMP9 antisense, 5′-CCA CCT GGT TCA ACT CAC T-3′; CD44 sense, 5′-GGT GAA CAA GGA GTC GTC-3′; CD44 antisense, 5′-TTC CAA GAT AAT GGT GTA GGT G-3′; SPP1 sense, 5′-CAG TGA TTT GCT TTT GCC-3′; SPP1 antisense, 5′-AGA TGG GTC AGG GTT TAG-3′; MMP7 sense, 5′-GGG ACT CCT ACC CAT TTG-3′; MMP7 antisense, 5′-CCA GCG TTC ATC CTC ATC-3′; CXCR4 sense, 5′-GGA AAT GGG CTC AGG G-3′; CXCR4 antisense, 5′-GAT GGA GTA GAT GGT GGG-3′; CDH1 sense, 5′-ATT GAA TGA TGA TGG TGG AC-3′; CDH1 antisense, 5′-GCT GTG GAG GTG GTG AGA-3′; GAPDH sense, 5′-CTC CTC CAC CTT TGA CGC-3′; and GAPDH antisense, 5′-CCA CCA CCC TGT TGC TGT-3′; PHB2 sense, 5′-GCT GGA CTA CGA GGA ACG-3′; PHB2 antisense, 5′-CTG TGA GGC ATT GAA CTT-3′; Vimentin sense, 5′-TTG AAC GCA AAG TGG AAT-3′; Clusterin (CLU) sense, 5′-ACG AGA AGG CGA CGA TGA-3′; CLU antisense, 5′-CTG GGA GGG GTT GTT GGT-3′; Gelsolin (GSN) sense, 5′-ACG ATG CCT TTG TTC TGA-3′; GSN antisense, 5′-TCT GGC TCG CTG CCT TCT-3′; S100A11 sense, 5′-CCT GAT TGC TGT CTT CC-3′; S100A11 antisense, 5′-AGG GTC CTT CTG GTT CT-3′;Vimentin antisense, 5′-AGG TCA GGC TTG GAA ACA-3′.

### iTRAQ labeling coupled with LC–MS/MS

#### Protein sample preparation and iTRAQ labeling

Spheroids were homogenized in lysis buffer (7 M urea, 2 M thiourea, and 1% cocktail proteinase inhibitor) using Precellys bead beating homogenizer (Bertin Technologies, France) for total protein extraction. The quality and concentration of the total proteins were measured using SDS–PAGE and a 2D quantification kit, respectively. Briefly, 100 µg of protein in each sample was precipitated with ice-cold acetone overnight at −20°C. The protein pellets were dissolved, reduced, denatured, blocked, and digested with sequencing-grade modified trypsin (Sigma; ratio of protein to enzyme, 20∶1; 37°C, overnight) as described in the iTRAQ protocol. The peptides were labeled as follows: Day 0, iTRAQ 117; Day 5, iTRAQ 118; Day 10, iTRAQ 119; and Day 15, iTRAQ 121. The iTRAQ-labeled peptides were mixed and dried using a rotary vacuum concentrator (Christ RVC 2–25; Osterodeam Harz, Germany), and the iTRAQ labeling experiment was independently carried out in triplicate.

#### Off-line 2D LC–MS/MS

The labeled peptides were desalted using a Sep-Pak Vac C18 cartridge(Waters, Milford, USA) and then fractionated by a strong cation-exchange (SCX) chromatography - on a 20AD HPLC system (Shimadzu, Japan) using an SCX column (polysulfoethyl column, 2.1 mm×100 mm, 5 um, The Nest Group, Inc. USA). Mixed peptides were eluted using a linear binary gradient of 0–45% buffer B (350 mM KCl, 10 mM KH2PO4 in 25% ACN, pH 2.6) in buffer A (10 mM KH2PO4 in 25% ACN, pH 2.6) at a flow rate of 200 µL/min for 60 min. A total of 28 fractions were collected. Each SCX fraction was dried, dissolved in buffer C (5% (v/v) acetonitrile and 0.1% formic acid), and analyzed on a QSTAR XL LC-MS/MS system (ABI, USA) with an RPLC column (ZORBAX 300SB-C18 column, 3 um, 75 um×150 mm, Microm, Auburn, CA). The RPLC gradient was 5% to 35% buffer B (95% acetonitrile, 0.1% formic acid) in buffer A (5% ACN, 0.1% formic acid) at a flow rate of 0.3 uL/min for 90 min.

#### Data analysis

All SCX fractions were analyzed twice using a QSTAR XL LC–MS/MS system (Applied Biosystems, USA). Data were acquired automatically using Analyst QS 1.0 Service Pack 8 (ABI/MDS SCIEX, Concord, Canada). Analysis survey scans were acquired from 400–1800 m/z and the 6 most intense peaks over 30 counts with a charge state of 2–4 were selected for MS/MS scan acquired from 100–2000 m/z. Other mass spectrometry parameters were set as following: curtain gas was set to 10, nitrogen was used as the collision gas, ionization tip voltage was set to 2,800 V and a rolling collision energy (CE) was applied for peptide fragmentation.

Protein Pilot software (version 3.0) was used for data processing and database searching. The following parameters were set in the searching: sample type, iTRAQ (4-plex peptide labeled); enzyme, trypsin; cycteinemodification: methylmethanethiosulfate; no special factors; biological modification; searching effort, thorough identification search. All proteins were identified at ≥95% confidence level. In addition, protein score threshold cutoff determined by Protein Pilot was set to 1.3 (Prot Score). At least one unique peptide with 95% confidence was considered for protein quantification. For iTRAQ quantitation, the peptide for quantification was automatically selected by the Pro Group algorithm (at least one peptide with 99% confidence) to calculate error factor (EF), and *P*-value. The true value for the average ratio was expressed and calculated as an EF (*EF* = 10 at 95% confidence level). An *EF* of <2 was set to satisfy quantification quality. In addition, a *P*-value of <0.05 was considered significant for protein quantification. Ratios of the 117, 118, 119, and 121 signature mass tags generated by MS/MS fragmentation from the iTRAQ- labeled peptides were calculated using Protein Pilot (version 3.0, ABI, USA) in Analyst. To designate significant changes in protein expression, fold changes of >1.2 or <0.83 were set as cutoff values. “Auto” bias correction was used to reduce artificial errors.

The differential proteins were further analyzed in the context of Gene Ontology (GO) biological process using the molecule annotation system 2.0 (MAS 2.0, http://bioinfo.capitalbio.com/mas3/) software (CapitalBio, Beijing, China).

### Western blot

The total protein (50 ug) extracted from the spheroid was resolved in 10% SDS-PAGE gels, followed by transferring onto PVDF membrane (Millipore, USA). After blocking in buffer (0.5% Tween-20 in TBS, and 5% w/v dried skimmed milk) for 1 h at room temperature, the membrane was incubated with the diluted primary antibody overnight at 4°C. Subsequently, it was washed with TBST (TBS with 0.5% Tween-20) and reacted with a HRP-conjugated secondary antibody (1∶10,000) for 1 h at room temperature. Finally, the target band was visualized using an ECL plus detection system. Primary antibodies used in the study were diluted as the following: ICAM-1(1∶500, Epitomics, Burlingame, CA, USA), ANXA1 (annexin A1, 1∶1000, Proteintech Group, Chicago, IL, USA), CK18 (1∶1000, Cell Signal Technology, Boston, MA, USA), FTL (ferritin, 1∶500, Proteintech Group, Chicago, IL, USA), GSN (Gelsolin,1∶2000, Epitomics, Burlingame, CA, USA), HSP90 (1∶500, Proteintech Group, Chicago, IL, USA), PCNA(1∶1000, Abcam, Boston, MA, USA), Vimentin (1∶1000, Epitomics, Burlingame, CA, USA) and beta-actin (1∶1000, HuaAn Biotechnology, Hangzhou,China).

### Statistical analysis

Data analysis was performed with SPSS 15.0 (SPSS, Chicago, IL, USA). Quantitative variables were expressed as mean ± SD (standard deviations) and analyzed using the Student's *t*-test. A two-sided *P* value of <0.05 was considered statistically significant.

## Results

### Dynamic expression patterns of invasion/metastasis-associated genes during the development of an *in vitro* HCC invasion model

**Figure 1:**
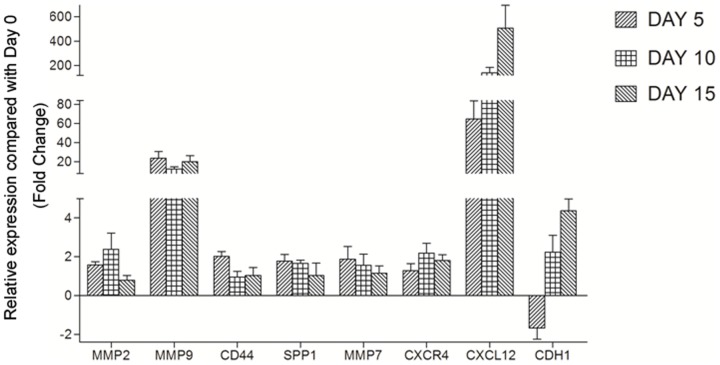
Expression patterns of eight invasion/metastasis-associated genes (MMP2, MMP7, MMP9, CD44, SPP1, CXCR4, CXCL12, and CDH1). Time course analysis showed remarkable, dynamic alterations in invasion/metastasis gene expression during the development of the HCC invasion model.

An *in vitro* HCC invasion culture model developed in our previous study could adequately mirror different pathological states of HCC invasion [16]. According to the pathological and morphological changes observed, day 5, in which HCC cells attached to the liver tissue fragment or slightly invaded it, and day 10, in which cells clearly invaded the liver tissue fragment, were defined as early stages of HCC invasion. By contrast, day 15, in which HCC cells formed tumor foci on the liver fragment, was defined as a late stage of invasion. Expression patterns of eight known invasion/metastasis-associated genes, including MMP2, MMP7, MMP9, CD44, SPP1, CXCR4, CXCL12, and CDH1, were used to evaluate dynamic alterations during the development of HCC invasion model (Fig. 1). Compared with those of the control (day 0), the expressions of MMP9, MMP7, and CD44 increased whereas that of CDH1 (E-cadherin) significantly decreased at early stages of invasion (day 5). The expressions of MMP2 and SPP1 were upregulated at early and middle stages of HCC invasion (days 5 and 10). Expressions of CXCL12 and CXCR4 were continually upregulated during the entire process of HCC invasion (days 5, 10, and 15). These results demonstrate remarkable, dynamic alterations in invasion/metastasis gene expression occurring along with the invasion process of HCC and indicate that HCC cells possess different invasion capabilities at different pathological states of invasion.

### Protein identification and categorization of common differential proteins

**Figure 2:**
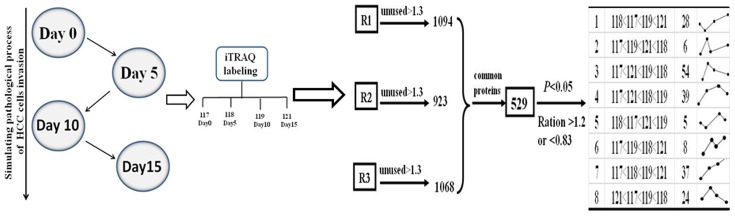
Schematic diagram showing the workflow of the current iTRAQ-based study using an *in vitro* HCC invasion culture model. Proteins from co-culture spheroids obtained at different time points (days 0, 5, 10, and 15) were labeled with iTRAQ tags (117, 118, 119, and 121). An average of 1,028 proteins (mean 1028±92; range, 923–1094) were identified in three independent experiments. A total of 529 common differential proteins were categorized by K-means clustering.

**Figure 3:**
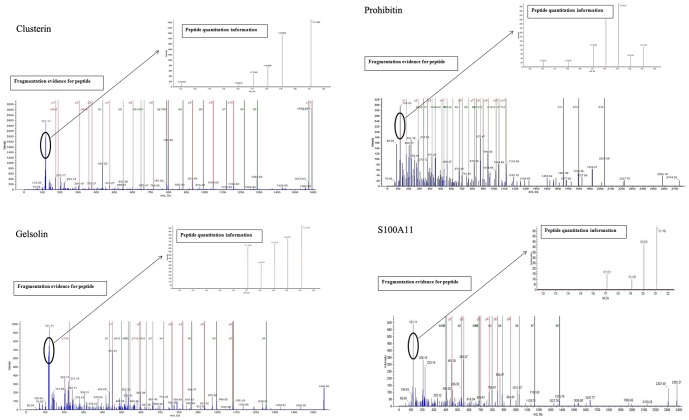
The MS/MS spectrums of representative differentially expressed proteins (Clusterin, Prohibitin, Gelsolin, and S100A11). The ion assignments are as follows: day 0, iTRAQ 117; day 5, iTRAQ 118; day 10, iTRAQ 119; day 15 iTRAQ 121.

**Figure 4:**
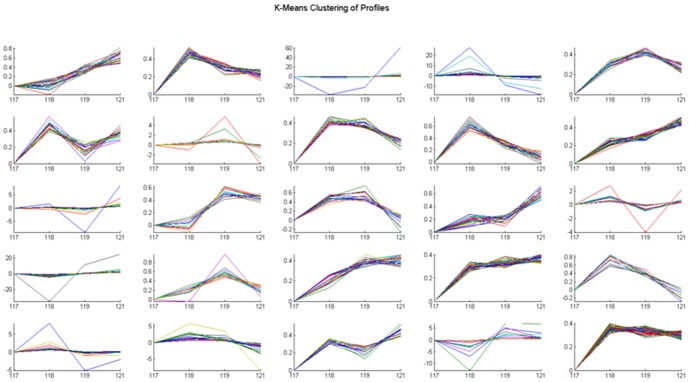
K-means clustering analysis of 25 expression patterns with different trend plots according to the relative expression levels of proteins at different time points. iTRAQ tags 117, 118, 119 and 121 represent day 0, day 5, day10, and day 15, respectively.

**Figure 5:**
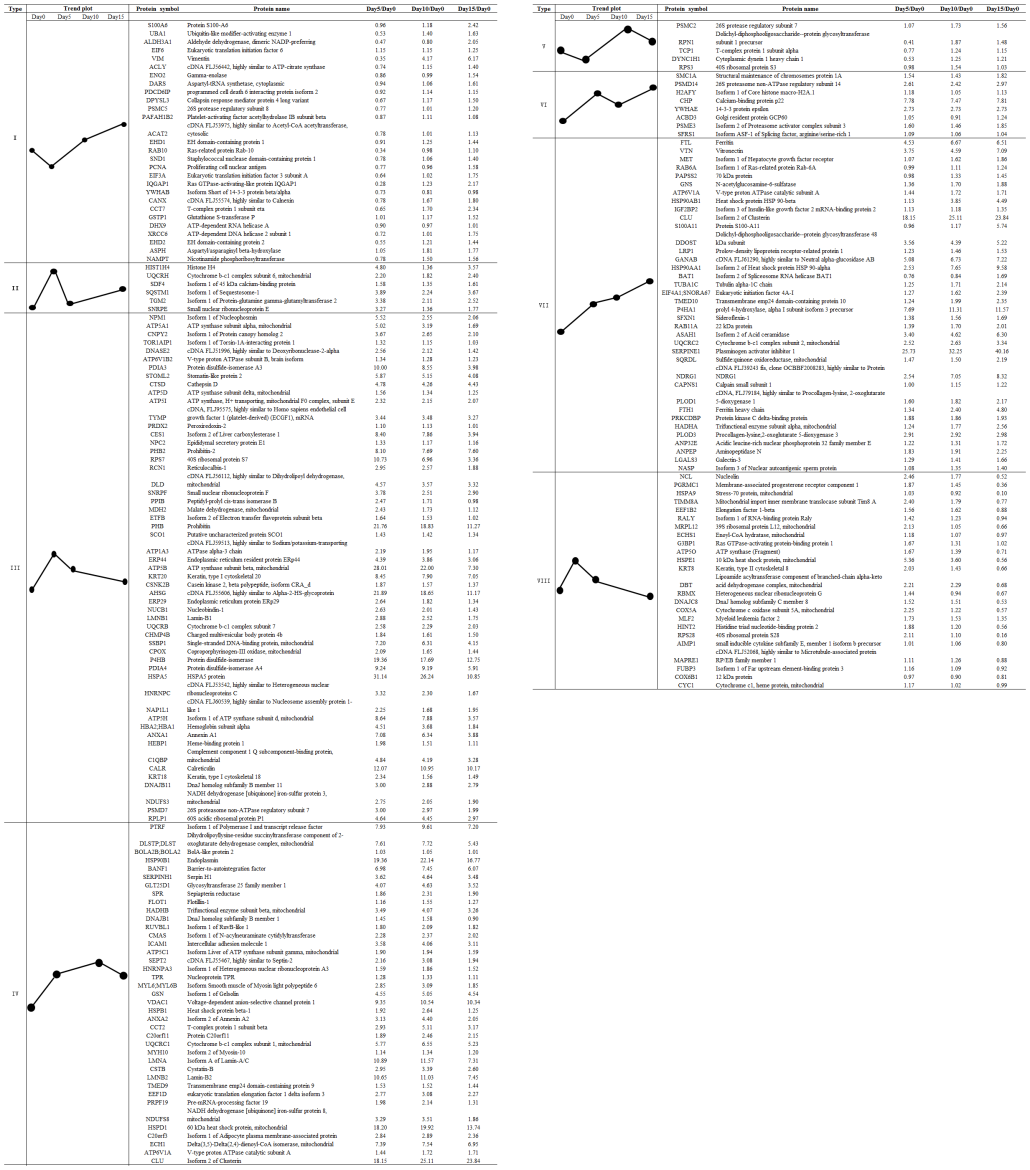
Eight typical expression patterns including 201 differential proteins during *in vitro* HCC invasion. Proteins obtained from the co-culture spheroids at different time points are labeled as Day 0, Day 5, Day 10, and Day 15.

**Figure 6:**
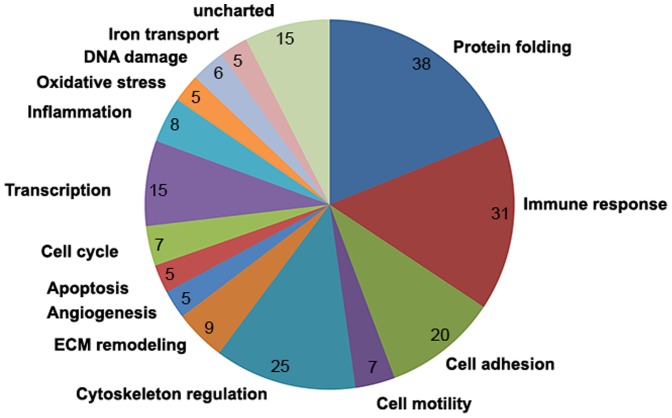
Molecular function classification of 201 differentially expressed proteins. Categorization showed that 32.8% (66/201) of differential proteins were associated with cell adhesion, cytoskeleton regulation, cell motility, ECM remodeling, and angiogenesis.

An average of 1,028 proteins (mean 1028±92; range, 923–1094) was identified by iTRAQ labeling coupled with LC-MS/MS in three independent experiments (Figs. 2 and 3). Among these proteins, 529 common differential proteins (fold-change of >1.2 or <0.83, *P*<0.05) related to HCC invasion were clustered into 25 types of expression patterns based on their relative expression levels at different time points (Figs. 2 and 4, Table S1). Eight typical expression patterns comprising 201 differential proteins (Types I to VIII, Fig. 5) were highlighted because of their significant dynamic alterations during the early invasion of HCC. These alterations included upregulation of expression at early invasion stages but downregulation at the late invasion stages (e.g., MAPRE1, PHB2, cathepsin D, TGM2, peroxiredoxin-2, lamin-B1, annexin A1, etc.) and continual upregulation of proteins throughout the entire invasion process (e.g., vitronectin, met, clusterin, ICAM1, GSN, S100A11, Hsp90, calpain, galectin, etc.). These identified proteins implicated in the early invasion of HCC were dynamically altered as invasion proceeded. Molecular function classification of these proteins showed that most of the significant differentially expressed proteins (66/201, 32.8% of all proteins) were associated with cell adhesion, cytoskeleton regulation, cell motility, ECM remodeling, and angiogenesis, which suggests that they contribute to pathological processes during early invasion of HCC (Fig. 6).

### Validation of differential expression patterns of proteins during early HCC invasion

**Figure 7:**
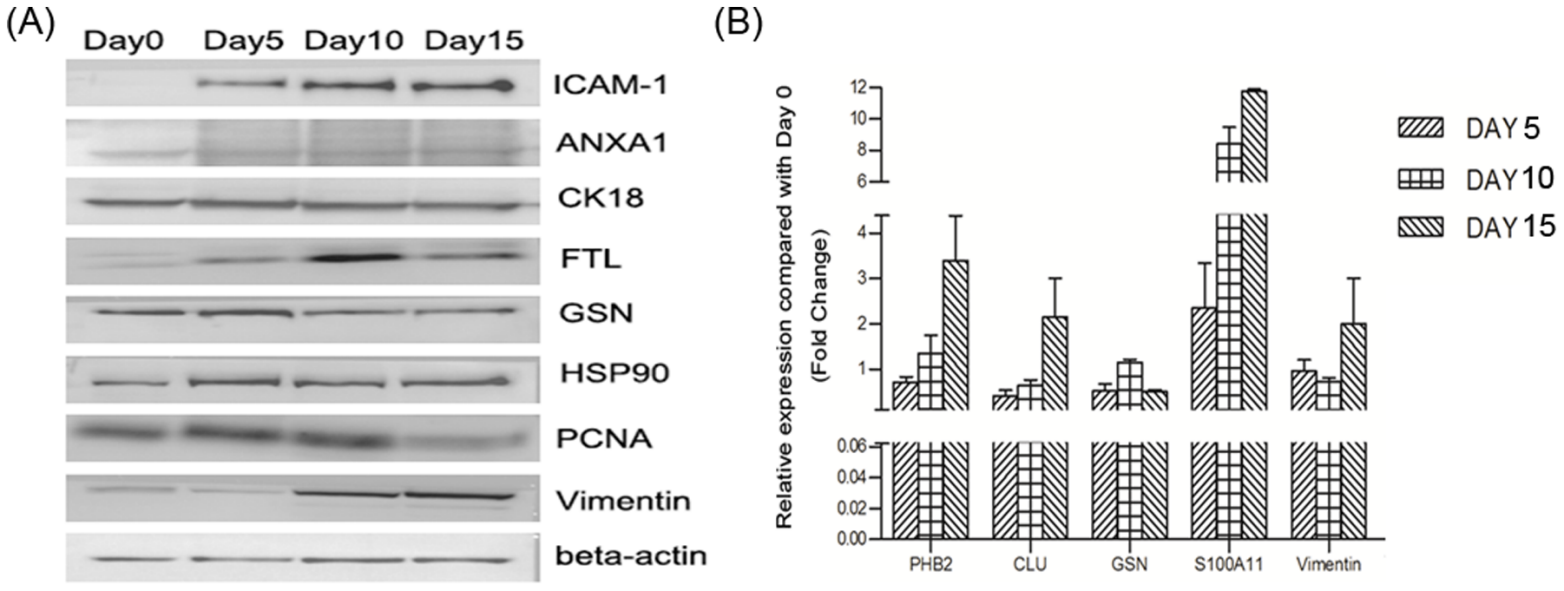
Dynamic alterations of some candidate differential proteins during early invasion of HCC were validated by Western blot (A) and qRT-PCR (B).

Dynamic alterations in differential protein expression related to HCC invasion (e.g. ICAM-1, ANXA1, CK18, FTL, GSN, HSP90, PCNA, Vimentin, PHB2, Clusterin, S100A11, and Vitronectin) were verified by Western blot (Fig. 7A) and quantitative RT–PCR (Fig. 7B), respectively. Dynamic changes of differential proteins validated by both Western blot and quantitative RT–PCR were all consistent with their expression modes acquired from quantitative proteomics.

## Discussion

Tumor cell invasion into the surrounding matrix is an early event of metastasis occurrence [17], [18]. Proteins that contribute to this early pathological process of invasion may be key molecular targets for early intervention of HCC invasion and metastasis. However, based on traditional cell experiment models and “after metastasis” comparative strategies, these proteins and their dynamic changes in expression can hardly be identified or defined at late stages of invasion. In contrast to previous reports on HCC metastasis, the current study mainly explores dynamic changes in key molecules during the early invasion process of HCC by means of a novel 3D HCC invasion culture model. This model was validated in our previous study [16] to better mimic the main pathological states of HCC invasion, including early invasion stages, such as attachment onto and invasion of HCC cells into liver tissue fragments, and late invasion stages, such as formation of tumor foci in the target tissue.

We evaluated dynamic alterations in invasion/metastasis gene expression using this HCC invasion culture model and found remarkable, dynamic alterations in invasion/metastasis gene expression that occurred along with the HCC invasion process. Some genes, such as MMP9, MMP7, CD44, and SPP1, were highly expressed at early invasion stages but eventually decreased at the late invasion stages. By contrast, the expression of CDH1 (E-cadherin) decreased at early invasion stages but increased at the late invasion stages. Some genes, such as MMP2 and CXCR4, were gradually upregulated at the early and middle stages of HCC invasion and then decreased at the late invasion stage. CXCL12 was continually upregulated during the entire invasion process. These results support the hypothesis that HCC cells can alter their invasion phenotypes and capabilities at different pathological stages of invasion. Thus, identification of candidate proteins during early invasion of HCC using this model and exploration of their dynamic expression patterns for early diagnosis and treatment of HCC are necessary.

iTRAQ-based quantitative proteomics is now widely applied in screening disease-associated differential proteins [19]. This technology enables comparative analysis of at most four different tissue samples with different pathological characteristics in one MS (mass spectrometry) experiment. Using this method, some proteins related to tumor metastasis have been successfully identified [5], [20]. However, little literature is found about the screening and identification of early invasion-associated HCC proteins. In the current study, a total of 1,028 differential proteins were identified in three independent experiments, and 529 common modulated proteins related to HCC invasion were screened for dynamic expression pattern analysis. These proteins were clustered into 25 types of expression patterns.

Eight typical expression patterns comprising 201 differential proteins, such as ICAM1, cathepsin D, vitronectin, Met, clusterin, and S100A11, were determined for future biological function analysis based on the following.

Generally, types II, III, and VIII proteins classified in Fig. 5, which increase at early stages of invasion but decrease at late stages, are ideal targets for early intervention. Considering their lower expression at the late stage of invasion or downregulation after completion of the invasion process, these identified proteins are not easily detected in clinical tumor tissue samples. To the best of our knowledge, most of these identified proteins, such as STOML2 (stomatin- like protein 2), SDF4, CES1, and PGRMC1, are not associated with HCC invasion and metastasis; thus, they may be considered unimportant by traditional research strategies. However, the biological functions of these proteins in early invasion deserve further investigation. Other proteins, such as MAPRE1, PHB2, cathepsin D, TGM2, peroxiredoxin-2, lamin-B1, and annexin A1, etc., have been reported to participate in HCC progression in other studies. MAPRE1 (EB1), also identified by proteomics analysis, is controlled by c-Myc, RhoA, and CDC42, all of which are linked to HCC malignancy, and shows prognostic prediction value for HCC [21]. PHB2 (prohibitin-2) increases the survival of HCC cells in hypoxic microenvironments [22]. Cathepsin D, which features proteolytic activity, serves as a prerequisite for cancer invasion, and its expression is significant in predicting HCC prognosis [23]. Why could these proteins be detected in the “after metastasis” samples? We speculate that among these “after metastasis” tumor tissues, several tumor samples with undetected aggressive or invasive events facilitate to discover this sort of candidate proteins.

Types IV, VI, and VII proteins classified in Fig. 5, which are continuously upregulated during the entire period of invasion, such as vitronectin, Met, Clusterin, ICAM1, GSN (Gelsolin), S100A11, Hsp90, Calpain, and Galectin, etc., are also potential valuable targets for disrupting the invasion process. Most of these proteins have been identified in clinical tissue specimens and confirmed to be associated with HCC invasion and metastasis. Vitronectin interacting with integrin *α*v*β*3 is involved in HCC metastasis. Aberrant Met activation promotes tumor growth, angiogenesis, and metastasis; in fact, many Met inhibitors have been developed for advanced HCC treatment [24]. Clusterin can induce EMT to promote HCC metastasis [25], [26]. ICAM1 is considered a marker of HCC stem cells, and its inhibitors suppress HCC tumor formation and metastasis [27]. GSN is involved in the Rack1/PI3K/Rac1 signaling pathway and affects HCC proliferation, migration, and invasion capacity [28]. Calpain is required for the invasive and metastatic potential of HCC cells and may be a drug target for preventing HCC metastasis [29]. Such findings further prove that this group of candidate proteins has important roles in the early invasion of HCC.

Types I and V proteins classified in Fig. 5, which are downregulated at early invasion stages but upregulated at the late invasion stage, are also involved in the process of HCC invasion and metastasis. Vimentin, S100A6, RAB10, and IQGAP1 are related to EMT, cytoskeletal dynamics, cell migration, and invasion. Vimentin, as an EMT-associated protein, is associated with HCC metastasis [30]. Network analysis has revealed that knockdown of vimentin can disturb the expression and stability of various cytoskeletal proteins, resulting in impaired HCC cell adhesion, motility, and metastasis [31]. The Ca^2+^-binding protein S100A6 mediates HBx-induced cell migration [32]. The small GTPase RAB10, which is frequently upregulated in HCC tissues [33], participates in vesicular transport. Increases in the scaffold protein IQGAP1 contribute to HCC tumorigenesis [34]; IQGAP1 can also integrate Rho GTPase and Ca^2+^/calmodulin signals with cell adhesion and cytoskeletal remodeling. This group of candidate proteins may be associated with the formation of metastatic tumor colonies after invasion.

We further selected some differential proteins representing different expression patterns and verified their dynamic expression patterns using Western blot and quantitative RT-PCR. Their dynamic changes of expression were all confirmed to be consistent with findings acquired from quantitative proteomics, suggesting that there exist the dynamic expression patterns for the identified proteins during early invasion of HCC. As such, the detailed biological functions of these target candidate proteins must be characterized in future studies.

Although there are many HCC cell types in previous studies, HCC cells with highly metastatic potential are always very rare. MHCC97H cells, established at our institute previously, have the malignant characteristics of strong invasion and metastasis shown in previous literatures [1], [6], [7], and are regarded as an ideal cell model for the study of HCC invasion and metastasis-related mechanism. Considering the major purpose of this study on dynamic expression of proteins during early invasion of HCC, highly invasion and metastasis characteristics become the preferred criterion for the selection of HCC cells. Additionally, a 3D HCC spheroid derived from MHCC97H has been established successfully in 3D culture system in our previous study [13]. Therefore, highly metastatic MHCC97H cells show great advantages over low metastatic HCC cells such as Hep3B, HepG2 in studying dynamic process of HCC invasion. Certainly, the defined pattern of protein profile from this study deserves to be validated in other HCC cell lines, which will be carried out in our future research.

In conclusion, the different expression patterns of candidate proteins identified from the invasion culture model demonstrate their diverse functions in the early invasion process of HCC. The dynamic expression patterns of candidate proteins observed during early invasion of HCC facilitate the discovery of new molecular targets for early intervention of HCC invasion and metastasis.

## Supporting Information

Table S1 Twenty-five expression patterns including 529 common differential proteins during *in vitro* HCC invasion. Proteins obtained from co-culture spheroids at different time points are labeled as Day 0, Day 5, Day 10, and Day 15.
(DOCX)
