# Supplementary material for: Dynamic Expression Patterns of Differential Proteins during Early Invasion of Hepatocellular Carcinoma
Source: PLoS One. 2014 Mar 10;9(3):e88543. doi: 10.1371/journal.pone.0088543 (PMC3948617; doi:10.1371/journal.pone.0088543)
Supplement: Table S1 — Twenty-five expression patterns including 529 common differential proteins during in vitro HCC invasion. Proteins obtained from co-culture spheroids at different time points are labeled as Day 0, Day 5, Day 10, and Day 15. (DOCX) [file pone.0088543.s001.docx]

**Table S1.** Twenty-five expression patterns including 529 common differential proteins during *in vitro* HCC invasion. Proteins obtained from co-culture spheroids at different time points are labeled as Day 0, Day 5, Day 10, and Day 15.
